# Comparative evaluation of methods for isolating small extracellular vesicles derived from pancreatic cancer cells

**DOI:** 10.1186/s13578-021-00550-3

**Published:** 2021-02-10

**Authors:** Jie-Min Wang, Yong-Jiang Li, Jun-Yong Wu, Jia-Xin Cai, Jing Wen, Da-Xiong Xiang, Xiong-Bin Hu, Wen-Qun Li

**Affiliations:** 1 Department of Pharmacy, The Second Xiangya Hospital, Central South University, Changsha, Hunan China; 2 Hunan Provincial Engineering Research Centre of Translational Medicine and Innovative Drug, Changsha, Hunan China; 3 Institute of Clinical Pharmacy, Central South University, Changsha, Hunan China

**Keywords:** Extracellular vesicles, Isolation method, Ultracentrifugation, Immunoaffinity capturing

## Abstract

**Background:**

Small extracellular vesicles (sEVs) are nanosized vesicles involved in cell-to-cell communication. sEVs have been widely studied for clinical applications such as early detection of diseases and as therapeutics. Various methods for sEVs isolation are been using, but different methods may result in different qualities of sEVs and impact downstream analysis and applications. Here, we compared current isolation methods and performed a comparative analysis of sEVs from supernatant of cultured pancreatic cancer cells.

**Methods:**

Ultracentrifugation, ultrafiltration and co-precipitation as concentration methods were firstly evaluated for yield, size, morphology and protein level of pellets. Then, isolate sEVs obtained by four different purification methods: size exclusion chromatography, density gradient ultracentrifugation, ultracentrifugation, and immunoaffinity capturing, were analysed and compared.

**Results:**

For the concentration process, ultracentrifugation method obtained high quality and high concentration of pellets. For the purification process, immunoaffinity capturing method obtained the purest sEVs with less contaminants, while density gradient ultracentrifugation-based method obtained sEVs with the smallest size. Proteomic analysis revealed distinct protein contents of purified sEVs from different methods.

**Conclusions:**

For isolating sEVs derived from supernatant of cultured pancreatic cancer cell line, ultracentrifugation-based method is recommended for concentration of sEVs, density gradient ultracentrifugation-based method may be applied for obtaining purified sEVs with controlled size, immunoaffinity capturing may be suitable for studies requiring sEVs with high purity but may loss subtypes of sEVs without specific protein marker.

## Introduction

Extracellular vesicles (EVs) are biological vesicles released by almost all types of cells. EVs have gained increasing interests over the last decade for their cell-to-cell communication properties. EVs have been emerging as attractive therapeutic tools for their content and their natural carrier role. Small EVs are able to be engineered as nano drug delivery vehicles due to their relatively small size and properties such as crossing the biological barrier, circulation stability and inherent targeting.

The methods for isolation of sEVs has been extensively studied. Conventional differential ultracentrifugation has been widely used, but may not be able to remove all contaminants [1, 2]. To obtain sEVs with small size, 0.22 μm filtration membranes were used in some studies to remove the microvesicles [3, 4], but the filtration membrane could not remove protein contaminants, which may mislead the study results [5, 6]. The International Society for Extracellular Vesicles (ISEV) advised that the qualities of EV from different isolation methods were different [7]. Besides, each isolation method may have disadvantages. Recently, it was reported that ultracentrifugation could not obtain EVs with high purity [1, 2], and may have problems such as clogging and trapping of vesicles [8], though the ultracentrifugation method could be improved by using a cushion of iodixanol [9]. Polymer co-precipitation-based methods are simple to perform but will precipitate large vesicles and contaminant proteins in the sample [10, 11]. Size exclusion chromatography-based method and density gradient ultracentrifugation-based may be effective but could also be time-consuming [12]. Application of immunoaffinity capture-based method was limited by target selection [13].

It seems to be reasonable to select the isolation method according to the demand for EV qualities. Generally, EV biomarker study demanded the purest EVs for exploring the relationship between EVs and diseases [14, 15], and EV therapeutic study demanded pure and large quantities of EVs [16]. Pancreatic cancer-derived sEVs have shown potentials for early disease detection [17] and therapeutic application [18]. However, there has been a lack of comparison of isolation methods of sEVs derived from pancreatic cancer cells.

**Fig. 1:**
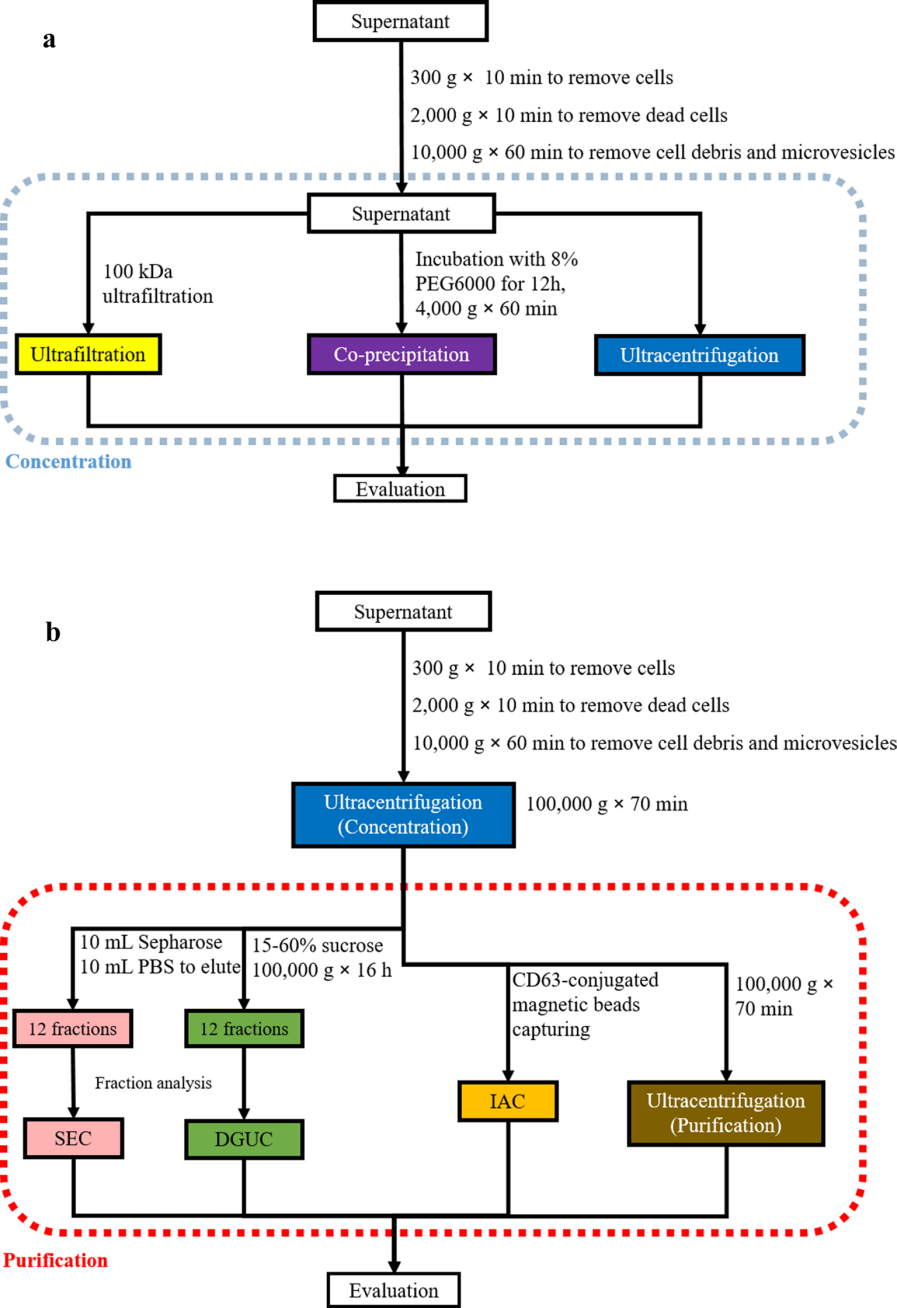
The workflow of the study. **a** Three different methods for concentrating sEVs from cell culture medium. **b** Four different methods for purification of sEVs after standard differential ultracentrifugation

This study aims to evaluate methods for isolation of pancreatic cancer-derived sEVs. A two-step isolation process was performed: concentration and purification. The concentration step aims to concentrate EVs from conditioned cell culture medium, while the purification step aims to purify crude sEVs. Human pancreatic Panc-1 cells, a widely used cell line for pancreatic cancer model, was used for production of sEVs [19]. A details isolation process of sEVs from Panc-1 cells was shown in Fig. 1.

## Methods

### Cell culture

The Panc-1 cell line was obtained from the ScienCell (USA). Cells were maintained in DMEM (Dulbecco’s modified Eagle’s medium, Thermo Fisher Scientific, USA) supplemented with 10% EV-depleted fetal bovine serum (obtained via ultracentrifugation at 160,000 g for 12h) (Thermo Fisher Scientific, USA). Cells were incubated at 37 °C with 5% CO_2_.

### Preparation of sEVs-containing medium

Before sEVs isolation, the supernatant from Panc-1 cells was handled as previously described [20]. Briefly, supernatant was centrifuged at 300 g for 10 min to remove cells, centrifuged at 2000 g for 10 min to remove the dead cells, and then centrifuged at 10,000 g (Thermo Fisher ST 16R, USA) for 1h to remove the cell debris and microvesicles [21]. The remaining supernatant was sEVs-containing medium.

### Preparation of crude sEVs

The following methods were separately used for preparation of crude sEVs (Fig. 1a): (1) Ultracentrifugation (UC): 50 ml of sEVs-containing medium was centrifuged at 100,000 g (SW32Ti, Beckman Coulter XPN-100 Ultracentrifuge, USA) for 70 min at 4 °C, and the pellet was re-suspended in 200 μL of PBS [21]; (2) Ultrafiltration (UF): 50 ml of sEVs-containing medium was concentrated using a 100 kDa ultrafiltration centrifugal tube (Merck Millipore, USA), PBS was added to re-suspend the concentrate, resulting in a final volume of 200 μL [22]; (3) Co-precipitation (Co-P): 50 mL of 8% PEG 6000 (Solarbio, China) solution was prepared and then mixed with 50 ml of sEVs-containing medium at 4 °C for 12 h, and then centrifuged at 4000 g for 60 min. The pellet was re-suspended in 200 μL of PBS. Crude sEVs were stored at −80°C (within 2 days.) before further purification.

### Purification of sEVs

Based on the results of evaluation of pellets after concentrating sEVs, ultracentrifugation was used for concentrating sEVs for further purification of sEVs. Four methods were used for purifying crude sEVs (Fig. 1b): (1) Density gradient ultracentrifugation (DGUC): 200 μL of crude sEVs was loaded onto the top of a 12 mL discontinuous sucrose (Solarbio, China) gradient solution (15%, 20%, 25%, 30%, 40%, 60% sucrose in PBS, 2 mL for each gradient solution) and then centrifuged at 100,000 g for 16 h at 4 °C. 12 fractions (1 ml) were collected for each gradient [23]. (2) Size exclusion chromatography (SEC): Sepharose CL-2B (Solarbio, China) was loaded into an injector (10 mL), with cotton blocked at the bottom. 200 μL of crude sEVs was loaded onto the top of the column. Then the column was eluted by PBS, and each 1mL of eluate was collected for 12 sequential fractions [24]. (3) UC: 200 μL of crude sEVs was washed in PBS. Then the solution was centrifuged at 10,000g, 4 °C for 70 min. The pellet was re-suspend by 200 μL of PBS. (4) Immunoaffinity capture (IAC): Magnetic beads (BeaverBeadsTM Protein A/G immunoprecipitation kit, Beaver, China) were washed and then activated by incubating with 100 μL of anti-CD63 antibody (50 μg/mL, ab134331, abcam, UK) for 15 min. After magnetic separation, the anti-CD63-conjugated beads were incubated with 200 μL of crude sEVs at 25 °C for 1 h. The sEVs-beads complexes were separated by a magnet and eluted and re-suspended in 40 μL of PBS. Purified sEVs were stored at -80°C within two days before analysis.

### Analysis of fraction from DGUC and SEC

Total protein level in each fraction was determined by using a BCA Protein Assay Kit (MultiSciences Biotech Co., China). The level of CD63 (CSB-E14107h) and CD9 (CSB-EL004969HU) in each fraction was determined by using an enzyme-linked immuno-sorbent assay (ELISA) kit (CUSABIO Biotech Co. Ltd., China). After analysis, fractions 6, 7, 8, 9, 10 from DGUC were mixed used for further analysis, while fractions 9, 10 from SEC were mixed and used for further analysis.

### Characterization and analysis of crude sEVs and purified sEVs

#### Nanoparticle tracking analysis (NTA)

For crude sEVs, samples were diluted 100 times with PBS before NTA. For purified sEVs, samples obtained via IAC was diluted 125 times, samples obtained via SEC was diluted 2.5 times, samples obtained via UC was diluted 25 times, while samples obtained via DGUC was not diluted before NTA (NS300, Malvern, UK). The RR of particles was calculated as the following formula. The assay was repeated 3 times.

1 $$RR\, of\, particles =\frac{number\, of\, particles\, in\, purified\, sEVs}{number\, of\, particles\, in \,crude\, sEVs}$$

#### Transmission electron microscopy

10 μL of samples was dropped onto an ultrathin carbon film-coated 400 mesh copper grid and washed with PBS for two times. After drying excess liquid, the EVs-coated grid was stained by phosphotungstic acid (1%) and then washed with PBS for two times and then dried and imaged with a multipurpose field emission transmission electron microscope (TEM, JEM-1200EX, JEOL Ltd., Japan).

#### Protein level

The protein level was determined by using a BCA Protein Assay Kit. The Protein Recovery rate (RR) of the purification process was calculated as the following formula. The assay was repeated 3 times.

2 $$RR \,of\, protein=\frac{Total\, protein\, in \,purified \,sEVs}{Total \,protein \,in \,crude\, sEVs}$$

#### Western blotting

5× SDS-PAGE Loading Buffer (New cell and Molecular Biotech Co., Ltd, China) was added into the sample. The sample was kept at 100 °C for 10 min. 6.02×10 [9] EVs were loaded on each well in 12% SDS-PAGE (Lianke Bio, China). After the electrophoresis, the proteins were transferred to PVDF membrane (Millipore, USA). The membrane was blocked with 5% milk solution for 1.5 h, followed by incubation with anti-CD63 (ab134045, abcam, UK), anti-CD81 (GB111073, Servicebio, China), anti-CD9 (ab92726, abcam, UK), anti-CD47 (ab108415, abcam, UK), anti-GAPDH (AF7201, Affinity, China), and anti-ago 1(#9388, Cell Signaling, USA). The PVDF membrane was washed and then incubated with Peroxidase-conjugated Goat anti-Rabbit IgG (ZSGB-Bio, China). The membrane was incubated with ECL luminescent (New cell and Molecular Biotech Co., Ltd, China) for 3 min for detection.

#### Coomassie brilliant blue staining

5× SDS-PAGE Loading Buffer (New cell and Molecular Biotech Co., Ltd, China) was added into the sample. The sample was kept at 100 °C for 10 min. 6.02×10^9^ EVs were loaded on each well in 12% SDS-PAGE (Lianke Bio, China). After the electrophoresis, the gel was incubated with 20 mL of working solution (0.0025% Coomassie brilliant blue, 45% methanol, 10% glacial acetic acid) (Solar bio, China) for 1 h, and then washed by elution solution (25% methanol, 8% glacial acetic acid) for 4 h.

#### ELISA

The level of CD63, CD81, TSG101, beta-actin, GAPDH, CD47 (CUSABIO Biotech Co. Ltd., China) and ago-1 (MyBiosource, Canada) in purified samples and in crude sEVs were determined by ELISA kits. The protein per EV was calculated by the following formula. The assay was repeated 3 times.

3 $$Protein\, per\, EV\,=\,\frac{total\, protein}{number\, of\, EVs}$$

#### Digestion of proteins

SDT solution (4% SDS, 100 mM Tris-HCl, pH 7.6) was added into the purified sEVs. The sample was incubated under boiling water for 15 min, followed by centrifugation at 14,000 g for 15 min. The supernatant was collected as protein sample. DTT (Sigma, USA, 43819-5G) was added in the protein sample to 100 mM. The sample was incubated under boiling water for 5 min, and then cooled to room temperature. 200 μL of UA buffer (8M Urea, 150mM Tris-HCl, pH 8.5) was added to the sample, followed by centrifuging at 12,500 g for 15 min using a 30 kDa ultrafiltration tube and centrifuged at 12,500 g for 15 min. Then, 100 μL of iodoacetamide (IAA) buffer (100mM IAA in UA) was added and kept at room temperature in darkness for 30 min. The sample was centrifuged at 12,500 g for 15min. 100 μL of UA buffer was added to the supernatant and then centrifuged at 12,500 g for 15 min again. 100 μL of 40 mM NH_4_HCO_3_ solution was added to the sample followed by centrifugation at 12,500g for 15 min. Then, 40 μL of Trypsin buffer was added (4μg Trypsin in 40 μL of 40 mM NH_4_HCO_3_) to the sample and incubated at 37°C for 16–18 h. The sample (in a filtration tube) was centrifuged at 12,500 g for 15 min, then 20 μL of 40 mM NH_4_HCO_3_ solution was added and centrifuged at 12,500 for 15 min to obtain the filtrate. A C18 cartridge (WAT023590, Waters) was used to desalinate. After freeze-drying, 40 μL of 0.1% methanol solution was added to the solid to re-solute the sample.

#### LC-MS/MS

Then sample was separated by Easy nLC (Thermo Fisher Scientific, USA). Solution A: 0.1% FA; Solution B: 0.1% FA, 80% ACN. Chromatographic column (Acclaim PepMap RSLC 50 μm × 15 cm, nano viper, P/N164943, Thermo Fisher Scientific) was balanced by 100% solution A. Velocity of flow was 300 nL/min. Gradient elution: 0–5 min, solution B 3%; 5–45 min, solution B 3%–28%; 45–55 min, solution B 28%–38%; 50–55 min, solution B 38%–100%; 55–60 min, solution B 100%.

The sample was analysed by Q Exactive (Thermo Fisher Scientific, USA). Analysis time was 1 h. The mode was positive ion mode. Range of parent ion was 350–1800 m/z. The resolution of mass spectrometry was 7000. AGC target was 3e^6. 1^ stage Maximum IT was 50 ms. Via full scan 10 MS2 scans were acquired. MS2 Activation Type was HCD. Isolation window was 2 m/z. The resolution of 2 stage mass spectrometry was 17,500. Microscan was 1. 2 stage. Maximum IT was 45ms. Normalized Collision Energy was 27eV.

Data-dependent acquisition was performed. The peptides database was Uniprot_HomoSapiens_20386_20180905, downloaded in http://www.uniprot.org. MaxQuant 1.5.5.1 was used to qualitative analysis. Label Free Quantitation was used for quantitative analysis.

### Statistical analysis

Data were presented as mean values ± SD. One-way analysis of variance (ANOVA) and students’ t test were performed at the significance level α = 0.05.

## Results

### Analysis of crude sEVs

**Fig. 2:**
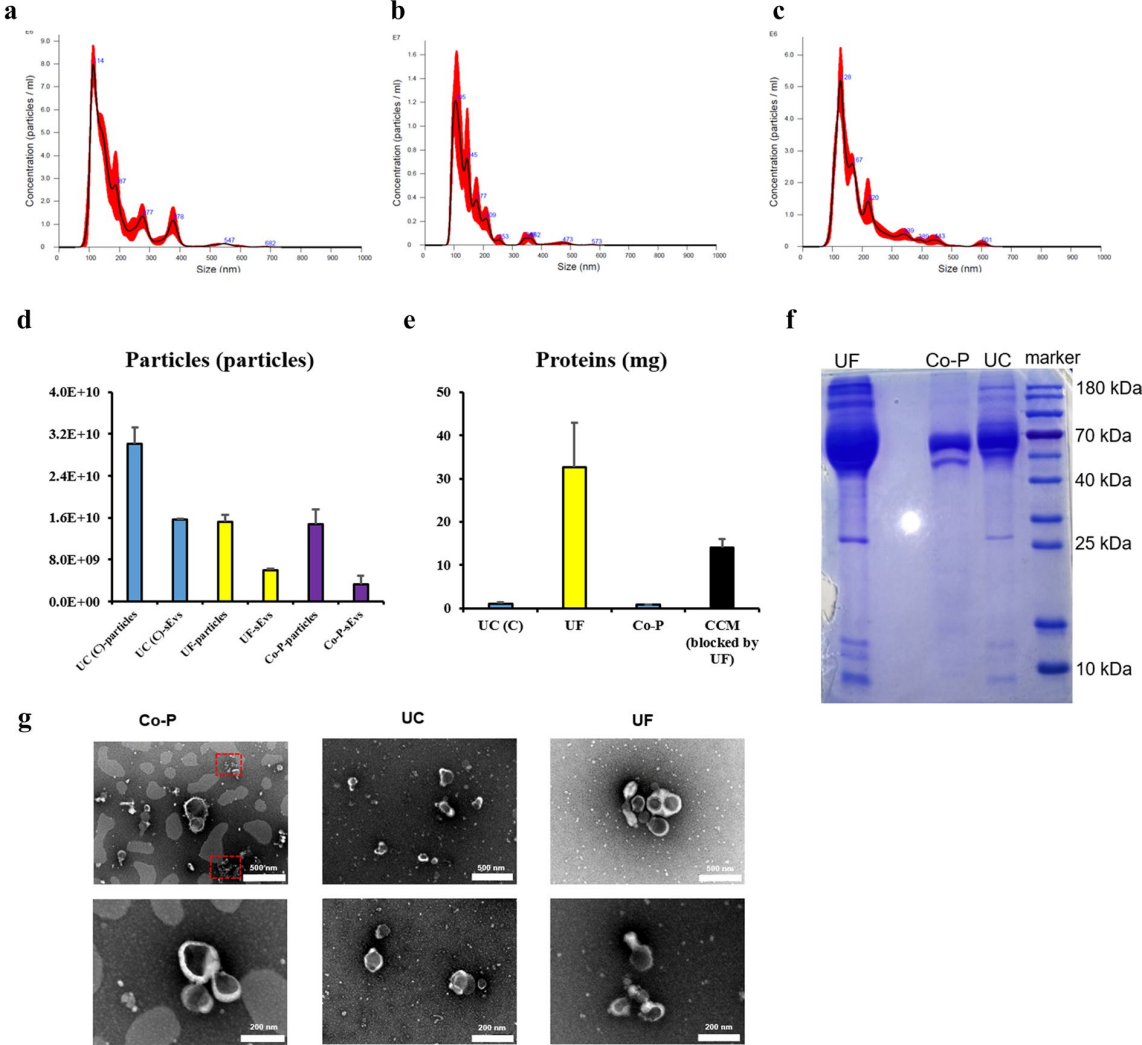
Evaluation of crude sEVs obtained via three different concentration methods. **a** Size distribution of pellets via ultrafiltration (n = 3). **b** Size distribution of pellets via ultracentrifugation (n = 3). **c** Size distribution of pellets via co-precipitation (n = 3). **d** The numbers of total particles and sEVs (30–150nm) in the pellets (n = 3). **e** The yield of the protein in pellets after concentration process (n = 3). **f** Coomassie brilliant blue staining of pellet. **g** TEM images of pellets. Contaminants of aggregated small particles (red frame) could be observed in the pellet obtained via co-precipitation method

Same volume (50 ml) of the supernatant was processed to compare concentration methods. Size distribution of crude sEVs obtained via UF, UC and Co-P were presented in Fig. 2a, b and c, respectively. sEVs obtained via UC showed a smaller size distribution than other two methods. All samples showed plenty of big particles, indicating that neither single method (UC, Co-P or UF) could obtain pure sEVs. Crude sEVs obtained via UC showed significantly more total particle number and small particle (30-150nm) number than UF and Co-P (Fig. 2d). sEVs obtained via UF showed significantly high protein levels than UC and Co-P as evaluated by BCA assay test (Fig. 2e) and coomassie brilliant blue staining (Fig. 2f), but the protein level in the control (fresh medium after UC) was also very high. TEM images for crude sEVs were presented in Fig. 2g. Big and small EVs could be observed for all crude sEVs groups. Besides, aggregation of unknown small particles (red frame) was found in crude sEVs obtained via Co-P. Based on the quality of crude sEVs, UC was used for crude sEVs collection for further purifications.

### Analysis of fraction from DGUC and SEC

**Fig. 3:**
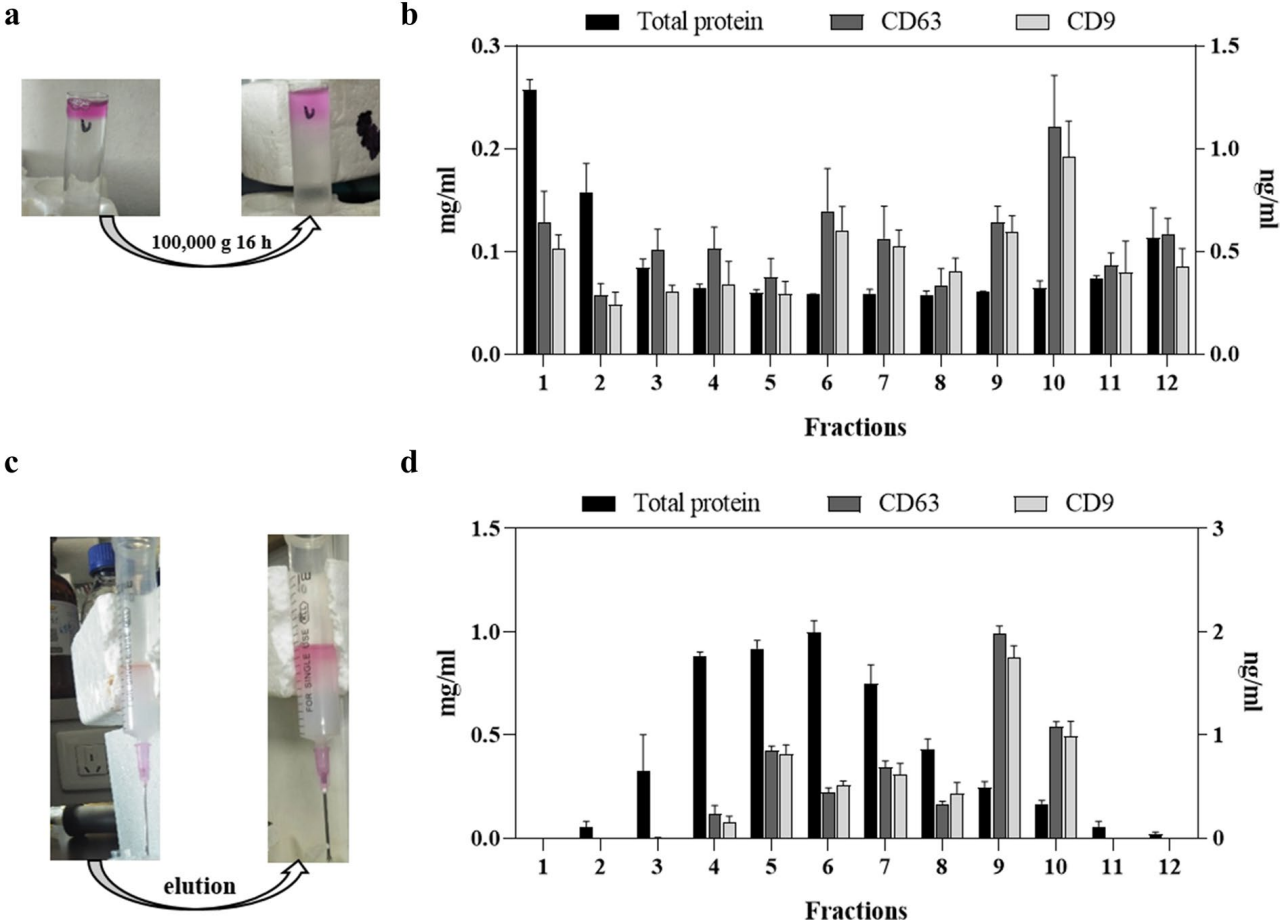
SEC purification process of the pellet obtained via ultracentrifugation. **a** The picture of the ultracentrifuge tube before and after ultracentrifugation. The border of the concentrate and ultracentrifugation rose solution became vague after ultracentrifugation. **b** Fraction analysis of DG ultracentrifugation (n = 3). Because of high CD63 concentration, the fraction 6, 7, 8, 9 and 10 were merged as the purified sample. **c** The picture of SEC, the EVs concentrate was eluted. **d** Fraction analysis of SEC (n = 3). Because of high CD63 concentration, the fraction 9 and 10 were merged as the SEC purified sample. * P < 0.05. Red color indicates the cell culture supernatant

Total protein, CD63 and CD9 levels in fractions from DGUC and SEC were shown in Fig. 3. Fractions with a relatively high level of CD63 and CD9 were collected as purified sEVs [24, 25]. CD63 and CD9 showed consistent levels in most fractions. Hence, fractions 6, 7, 8, 9 and 10 from DGUC were mixed as the purified sample (Figure 3a, b), fractions 9 and 10 from SEC were mixed as the purified sample (Figure 3c, d).

### Analysis of purified sEVs

**Fig. 4:**
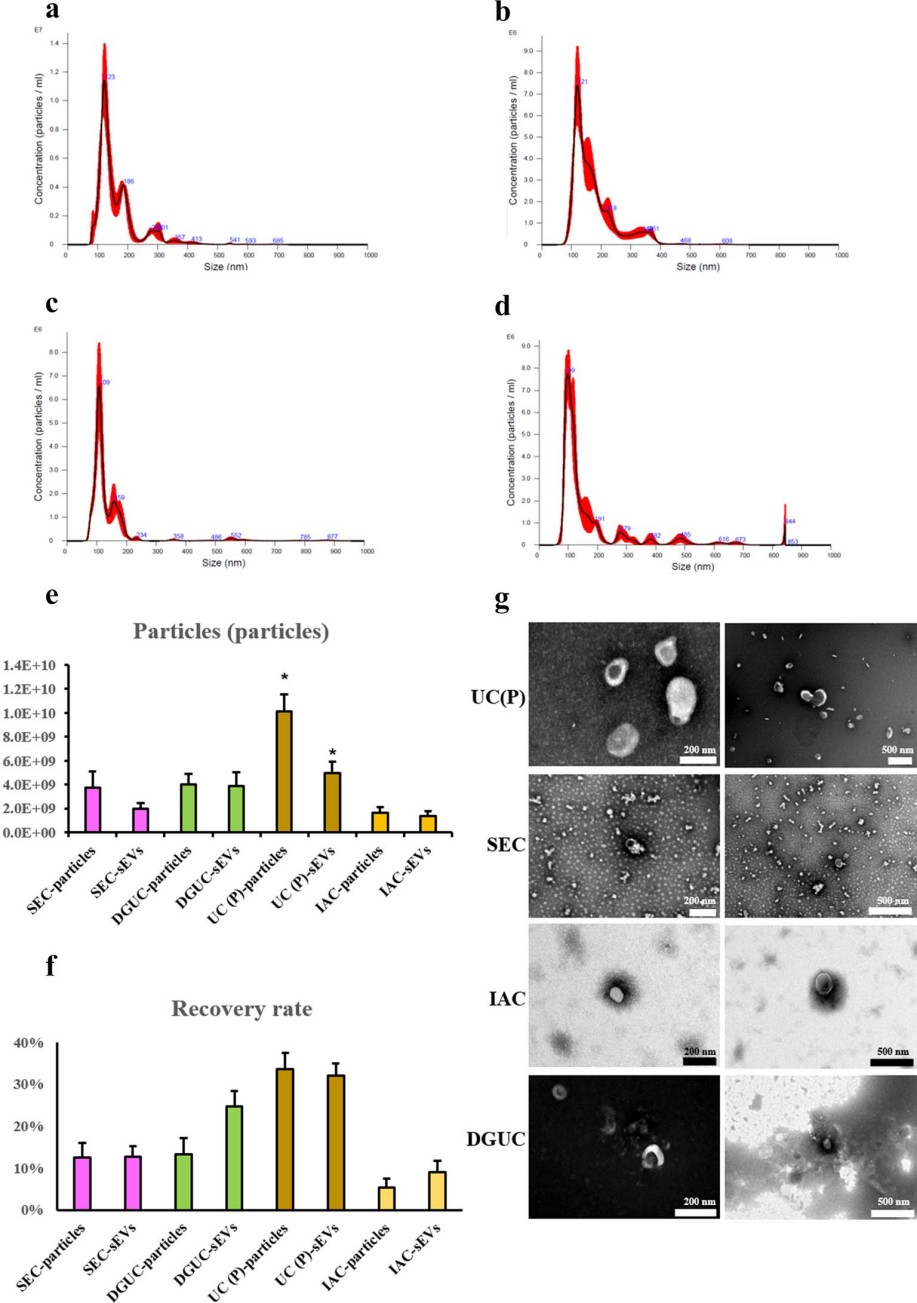
Evaluation of particles of the purified samples. **a** Size distribution of pellets via ultracentrifugation (n = 3). **b** Size distribution of pellets via SEC (n = 3). **c** Size distribution of pellets via IAC (n = 3). **c** Size distribution of pellets via DGUC (n = 3). **e** The numbers of total particles and sEVs (30–150 nm) in the pellets (n = 3). **f**RR of the particle and sEVs. **g** TEM images of the purified samples. **e** Dyed-gel. Compared of the concentrates, the purified samples showed lower protein content. * P < 0.05

The size distribution of purified samples via UC, SEC, IAC and DGUC were shown in Figure 4a–d, respectively. The big particles in sEVs for all samples were removed substantially. The size of the sample via DGUC showed the smallest size, and the sample via IAC showed a relatively small size with less microvesicles. The sample via UC showed more particle and sEVs numbers than other methods (Figure 4e). The sample via SEC showed the least particles and sEVs. Besides, samples via DGUC or IAC showed a relatively high proportion of sEVs in all particles (Figure 4e). The sample via UC showed the highest RR (Figure 4f). TEM images of purified sEVs were shown in Figure 4g. sEVs could be observed for all samples, while samples obtained via UC show relatively more particles. Samples obtained via IAC and DGUC showed clearer background under TEM. Contaminants were observed in samples obtained via SEC.

### Protein evaluation

**Fig. 5:**
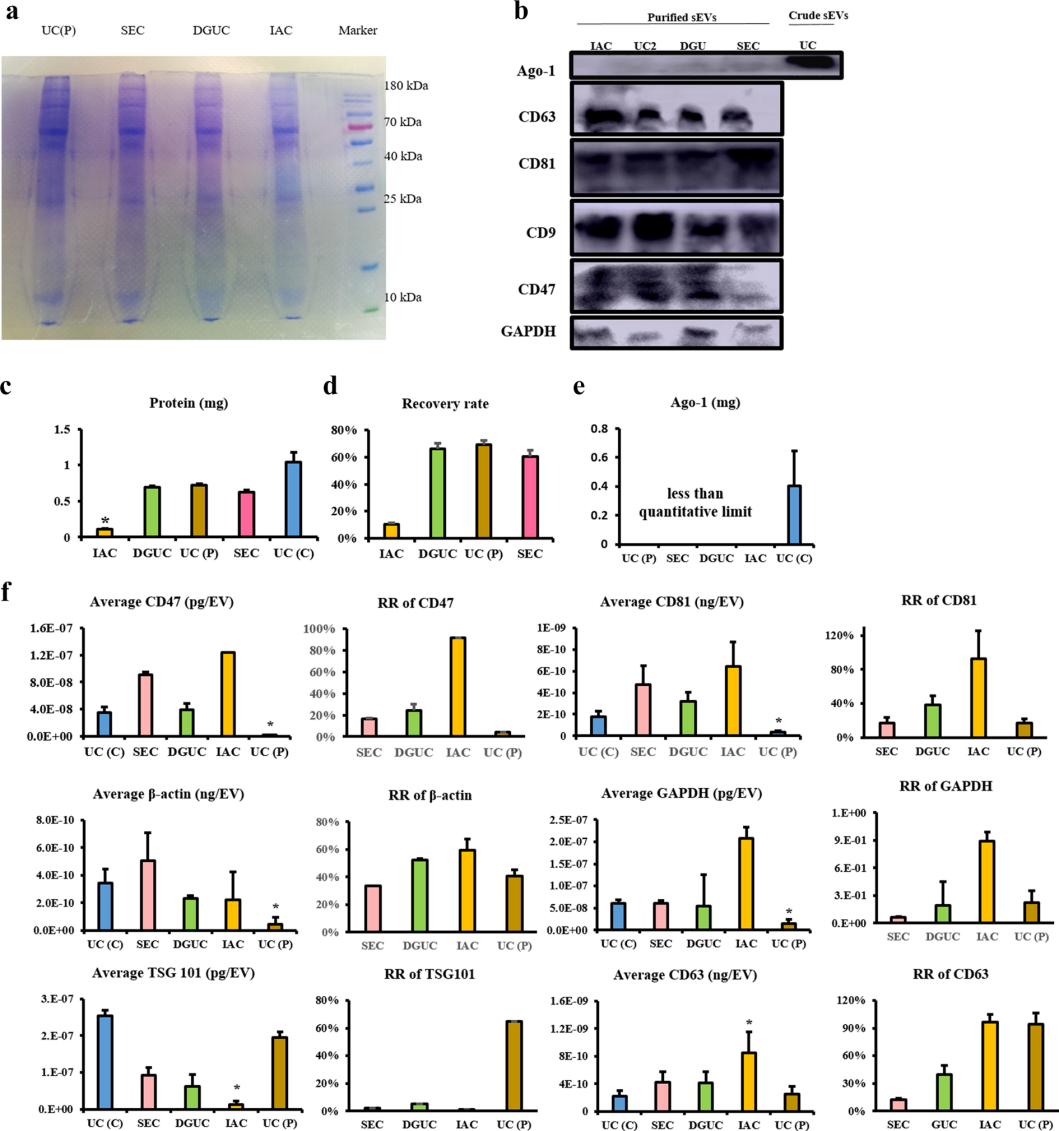
Evaluation of proteins of the purified samples. **a** Coomassie brilliant blue staining of pellet. **b** Western blot analysis of purified sEVs sample. **c** Levels of protein in purified sample (n = 3). **d** Recover rate of protein via different purification method. **e** Ago-1 level in purified samples. **f** Level of various proteins and recover rate in purified sample. * P < 0.05

Purified sEVs showed lower total protein levels for all methods than crude sEVs (Figure 5). The protein level of the sample via UC was higher than other methods as evaluated by BCA assay (Figure 5c) and coomassie brilliant blue staining (Figure 5a). The sample purified by IAC showed the least total protein level and recover rate (Figure 5c, d).

WB results were shown in Figure 5b. Three EVs marker proteins (CD81, CD63, CD9) and CD47 were detected in the purified samples. Compared to the crude sEVs, ago-1 as contaminant protein was significantly decreased in purified samples, which was consistent with the WB results (Figure 5e). Results of ELISA were summarized in Figure 5f. Samples purified by UC showed less CD47, CD81, GAPDH and β-actin. The sample via IAC showed the least TSG101. The sample via IAC had a higher CD63 level as the sample was isolated by anti-CD63-conjugated beads. RR of CD63 was almost 100% for IAC method, indicating that almost all particles expressing CD63 were extracted. For the samples purified by SEC and DGUC, levels of most proteins tested were similar.

### Proteomics

**Fig. 6:**
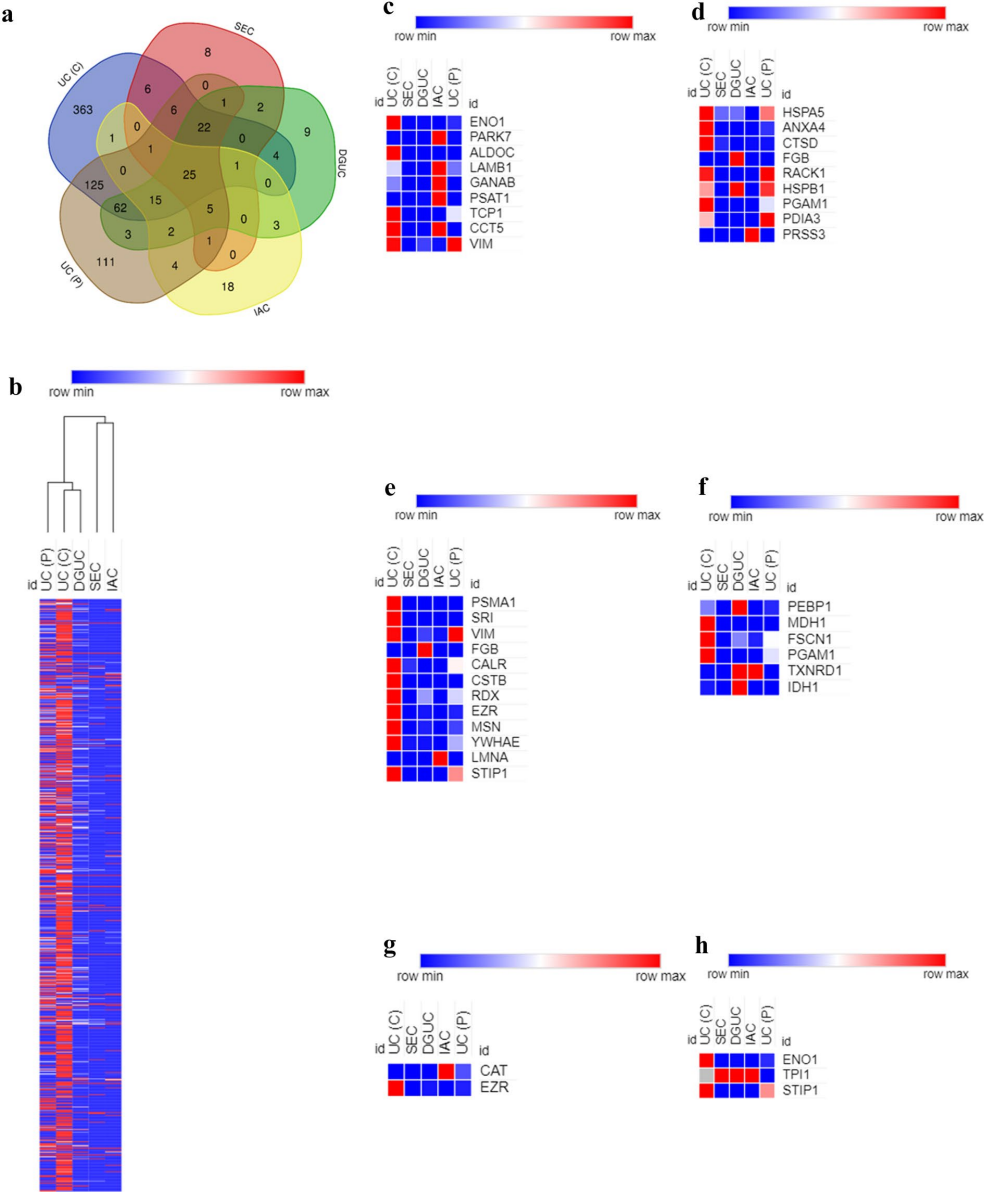
Proteomic analysis of sEVs. **a** Venn diagram for number of proteins in sample. **b** The heat map of all proteins of sEVs (n = 3). **c** Analysis of overexpressed proteins (pancreatic cancer tissue compared with normal adjacent pancreatic tissue) in sEVs. **d** Analysis of downexpressed proteins (pancreatic cancer tissue compared with normal adjacent pancreatic tissue) in sEVs. **e** Analysis of overexpressed pancreatic cancer metastasis-associated proteins in sEVs. **f** Analysis of downexpressed pancreatic cancer metastasis-associated proteins in sEVs. **g** Analysis of overexpressed pancreatic cancer drug resistance-associated proteins in sEVs. **h** Analysis of downexpressed pancreatic cancer drug resistance-associated proteins in sEVs

A total of 817 proteins were detected in proteomic study. There were 631 proteins in the crude sEVs sample via UC, 383 proteins in the purified sEVs sample via UC, 78 proteins in the sample via SEC, 154 proteins in the sample via DGUC and 76 proteins in the sample via IAC, 25 proteins were identified for all five groups (Fig. 6a). Heat map analysis of all proteins was summarized in Figure 6b. Purified sEVs obtained via UC showed significantly more protein content, which was consistent with our results of protein evaluation (Fig. 5). Reported potential protein biomarkers for cancer development [26], metastasis [27] and drug resistance [28] were also analysed in our proteomic study. Further analysis revealed distinct protein contents in samples (Fig. 6). High contents of both overexpressed and downexpressed proteins associated with metastasis and drug-resistance were detected in crude sEVs (Fig. 6c to h). Purified sEVs via IAC retained most pancreatic cancer-overexpressed (Fig. 6c), but not downexpressed (Fig. 6d), proteins (compared to normal adjacent pancreatic tissue). sEVs obtained via SEC showed the least contents of proteins associated with metastasis (Fig. 6e) and drug resistance (Fig. 6g).

## Discussion

Despite progress in technique, isolation of sEVs has been challenging. It would be wise to choose a strategy and develop a isolation specific protocol depending on experiment purpose. Three concentration methods and four purification methods for obtaining sEVs from cultured Panc-1 cells were evaluated in our study. A summarization of advantages, disadvantages and possible suggestions for isolation methods used in this study was shown in Table 1. For the concentration of sEVs, UC-based method showed high yield and purity of sEVs and was used to concentrate the sample before purification. For the purification of sEVs, IAC was effective and time-saving and yield purest sEVs among all methods evaluated, thus may be suitable for study demanding high purity of sEVs [14, 15]. DGUC method effectively produced the smallest and also purified sEVs with relatively high yield, thus may be suitable for obtaining sEVs with controlled size and large quantity [16, 29–31]. More importantly, sEVs obtained via DGUC method could avoid aggregation and precipitation [32] and were superior for cellular uptake [33].

**Table 1:** Summary of concentration and purification methods for isolating sEVs from pancreatic cancer cells

Process	Method	Advantages	Disadvantages	Possible suggestions	Recommendation
Concentration	UC	High yield	High equipment cost	NA	Suitable for concentrating medium
	Co-P	Convenient	Non-exosomal contaminants	NA	NA
	UF	Convenient	Non-exosomal contaminants	NA	NA
Purification	UC	High yield Feasibility	High equipment cost	Fully re-suspend the crude sEVs	NA
	SEC	High purity Up-scale isolation	Require methodological validation	Lengthen the column and increase the sepharose Slow down the flow rate Lower the temperature	NA
	DGUC	Small EVs High purity	Require methodological study Time-consuming centrifugation High equipment cost	Pilot test for exploring suitable gradient solutions	Suitable for therapeutic study
	IAC	Small EVs Time-saving High purity	Low yield Subtype of sEVs	Personalized customization depending on aim of study	Suitable for biomarker study

Co-P, Co-precipitation; DGUC, Density gradient ultracentrifugation; EV, Extracellular vesicles; IAC, Immunoaffinity capture; NA, Not available; SEC, Size exclusion chromatography; UC, Ultracentrifugation; UF, Ultrafiltration

In the presented study, the size, yield, morphology and protein were assessed for quality of sEVs. The size and yield of sEVs were crucial for therapeutic use, especially for drug delivery. It has been reported that smaller EVs could be uptaken by cells more efficiently [33]. The morphology showing the presence of EV was observed, sEVs obtained in this study was saucer-shaped under TEM images as reported [34]. CD63 was a marker for EVs and was used in IAC for purification [10, 35, 36]. Besides, in our fraction analysis, CD63 and CD9 levels were used to reflect EV-containing fractions, this method was also reported in previous studies [23, 25, 37]. However, for SEC, CD63 and CD9 may not fully represent the sEVs fractions as expected. Possibly, CD63 and CD9 were not exclusive to sEVs. Recent studies reported that microvesicles also expressed CD63 and CD9 [38, 39]. A combined strategy of more sEVs marker proteins such as TSG101 and CD81 may reflect the sEVs-containing fractions more accurately.

Our study included the majority of current sEVs isolation methods, except commercial kits and microfluidic-based methods. Because of unstable quality of the extracted EVs, high price and unknown solutions [40], commercial kits were not included in our study. The microfluidic technology was popular but not included in our study as the technique was mostly used for methodological studies, such as sEVs detection, instead of therapeutic application study, even if it was potentially available for isolation [41, 42].

In the presented study, an equal number of EVs particles was evaluated for each method in case of contaminant protein influencing the results. The western blot analysis showed inconsistent intensity of GAPDH as cytosolic protein in sEVs, suggesting that different isolation methods would result in inconsistent purity and yield of sEVs and possibly subtype of sEVs. The level of target protein could be weak as detected if equal protein, with a high amount of contaminant protein, was loaded onto each well in the gel. This may explain why the level of EV marker protein was weak in study [43].

The UC-based technique remains the most common method for sEVs isolation [44–46]. However, our study results demonstrated that DGUC and IAC methods produced sEVs with better purity than UC. Besides, the interpretation of results should be cautious when commercial sEVs isolation kits based on the Co-P method was used [40, 47]. The combination of several isolation methods may produce purer sEVs. Jeppesen et al. used ultracentrifugation-based technique, density gradient ultracentrifugation-based technique and immunoaffinity capture-based technique to purify sEVs [48]. But more isolation steps may produce fewer sEVs, and combined isolation protocols were often hard to follow as the strategy could be complicated and fussy. The technique for evaluation of sEVs has been advancing. Tian et al. splendidly used nanoflow cytometry to evaluate the quality of sEVs [40]. But nanoflow cytometry-based analysis of sEVs needs further refinement of methods. For better comparisons between groups and future replication, we applied basic characterization of sEVs isolated by different methods [25, 37, 49].

sEVs are a heterogenous group of vesicles [50]. Single EV may carry distinct proteins, isolation method based on EV marker may lose EV subtypes of potential interest. For example, anti-CD63 conjugated-beads could be used to obtain CD63-enriched EVs, but effects of other vesicles may be neglected during subsequent experiments. This may explain why several metastasis and drug resistance-associated proteins were not highly expressed in purified sEVs obtained via IAC. However, future basic and clinical studies are likely to provide valuable information regarding their heterogeneity and advance our understanding of biological functions, thus reveal and harness their potentials for disease detection and therapy.

### Conclusions

Current methods are useful for isolating sEVs. Recommendations for choosing sEVs isolating method depend on the aim of study. For isolation of Panc-1 cell-derived sEVs, UC was advised to concentrate the medium. For purification, DGUC method could obtain high amount of sEVs particles with controlled size, IAC method is effective for isolating sEVs with high purity, but may loss subtypes of sEVs via specific marker capturing.

## Data Availability

Not applicable.
